# Evaluation of STEMI Regionalization on Access, Treatment, and Outcomes Among Adults Living in Nonminority and Minority Communities

**DOI:** 10.1001/jamanetworkopen.2020.25874

**Published:** 2020-11-16

**Authors:** Renee Y. Hsia, Harlan Krumholz, Yu-Chu Shen

**Affiliations:** 1 Department of Emergency Medicine, University of California, San Francisco; 2 Philip R. Lee Institute for Health Policy Studies, University of California, San Francisco; 3 Section of Cardiovascular Medicine, Department of Internal Medicine, Yale School of Medicine, New Haven, Connecticut; 4 Department of Health Policy and Management, Yale School of Public Health, New Haven, Connecticut; 5 Center for Outcomes Research and Evaluation, Yale–New Haven Hospital, New Haven, Connecticut; 6 Graduate School of Defense Management, Naval Postgraduate School, Monterey, California; 7 National Bureau of Economic Research, Cambridge, Massachusetts

## Abstract

**Importance:**

Cardiac care regionalization, specifically for patients with ST-segment elevation myocardial infarction (STEMI), has been touted as a potential mechanism to reduce systematic disparities by protocolizing the treatment of these conditions. However, it is unknown whether such regionalization arrangements have widened or narrowed disparities in access, treatment, and outcomes for minority communities.

**Objective:**

To determine the extent to which disparities in access, treatment, and outcomes have changed for patients with STEMI living in zip codes that are in the top tertile of the Black or Hispanic population compared with patients in nonminority zip codes in regionalized vs nonregionalized counties.

**Design, Setting, and Participants:**

This cohort study used a quasi-experimental approach exploiting the different timing of regionalization across California. Nonpublic inpatient data for all patients with STEMI from January 1, 2006, to October 31, 2015, were analyzed using a difference-in-difference-in-differences estimation approach.

**Exposure:**

Exposure to the intervention was defined as on and after the year a patient’s county was exposed to regionalization.

**Main Outcomes and Measures:**

Access to percutaneous coronary intervention (PCI)–capable hospital, receipt of PCI on the same day and at any time during the hospitalization, and time-specific all-cause mortality.

**Results:**

This study included 139 494 patients with STEMI; 61.9% of patients were non-Hispanic White, 5.6% Black, 17.8% Hispanic, and 9.0% Asian; 32.8% were women. Access to PCI-capable hospitals improved by 6.3 percentage points (95% CI, 5.5 to 7.1 percentage points; *P* < .001) when patients in nonminority communities were exposed to regionalization. Patients in minority communities experienced a 1.8–percentage point smaller improvement in access (95% CI, −2.8 to −0.8 percentage points; *P* < .001), or 28.9% smaller, compared with those in nonminority communities when both were exposed to regionalization. Regionalization was associated with an improvement to same-day PCI and in-hospital PCI by 5.1 percentage points (95% CI, 4.2 to 6.1 percentage points; *P* < .001) and 5.0 percentage points (95% CI, 4.2 to 5.9 percentage points; *P* < .001), respectively, for patients in nonminority communities. Patients in minority communities experienced only 33.3% and 15.1% of that benefit. Only White patients in nonminority communities experienced mortality improvement from regionalization.

**Conclusions and Relevance:**

Although regionalization was associated with improved access to PCI hospitals and receipt of PCI treatment, patients in minority communities derived significantly smaller improvement relative to those in nonminority communities.

## Key Points

**Question:**

Is regionalization of the care of patients with ST-segment elevation myocardial infarction (STEMI) associated with widened or narrowed disparities in access, treatment, and outcomes for patients with STEMI between minority and nonminority communities?

**Findings:**

In this cohort study, regionalization was associated with significantly smaller improvement for residents of minority communities compared with patients from nonminority communities when admission to PCI hospital and treatment received were measured from regionalization, ranging from only 15% to 71% of what patients in nonminority communities experienced. Moreover, White patients in nonminority communities experienced the largest improvement in care, whereas Black and Hispanic patients in minority communities had little or no improvement when exposed to regionalization.

**Meaning:**

Patients in minority communities compared with those in nonminority communities derived smaller benefits when these were measured by access to PCI hospitals and receipt of PCI from regionalization.

## Teaser

This cohort study examines the extent to which disparities in access, treatment, and outcomes have changed for patients with ST-segment elevation myocardial infarction (STEMI) who are living in zip codes that are largely composed of Black or Hispanic individuals compared with patients in nonminority communities in regionalized vs nonregionalized counties.

## Introduction

Disparities in the treatment and outcomes of acute myocardial infarction for minorities have been widely documented.^1,2,3,4,5,6,7,8^ Studies have consistently shown that Black^7,9,10^ and Hispanic^11^ patients have lower rates of revascularization of any type after an acute myocardial infarction, and have significantly longer door-to-needle and door-to-balloon times,^7,12,13,14,15,16,17,18^ with some improvements in door-to-balloon times in more recent years.^18^ For ST-segment elevation myocardial infarction (STEMI) specifically, Black patients are more likely than White patients to experience an in-hospital stroke or major bleeding after a STEMI and have higher long-term mortality.^12,19^

Cardiac care regionalization, specifically for the care of patients with STEMI, has been touted as a potential mechanism to reduce systematic disparities^20,21^ by protocolizing the treatment of these conditions. Regionalization of STEMI care is associated with increased use of reperfusion therapy and faster time to treatment.^22,23^ The goal of STEMI regionalization has been to achieve the recommended interventions—specifically, percutaneous coronary intervention within 90 minutes from first medical contact for direct transport to a percutaneous coronary intervention (PCI)–capable facility and within 120 minutes from first medical contact for transfers^24,25^—by designating hospital capabilities and emergency medical services (EMS) bypass of facilities with lesser designation when appropriate.^26,27,28^

Although regionalization has the potential to improve rapid access to primary PCI,^28,29,30^ scientific innovations often disproportionately benefit the most advantaged patients,^31^ whether that be specific medical technology such as surfactant therapy for premature newborns^32^ or populationwide initiatives such as tobacco control.^33^ This phenomenon may also be true of regionalization because PCI hospitals tend to open in wealthier communities that serve patients with private insurance, where PCI services already exist.^34,35^ There are few studies that have specifically addressed how regionalization has affected these communities. Currently, the only 2 studies of this topic were based on treatment times for Black and White patients who received treatment before and after implementation of regionalization programs, and they had contradictory results.^36,37^ The important foundational studies to date have evaluated only those patients who received the treatment (went to the intervention hospital) rather than the overall population (those in the catchment area), and in general have been underpowered to detect potential mortality benefits.^1,29,30,36,38,39,40,41,42,43^

Accordingly, this study seeks to fill this gap by using a population-based approach and exploiting the natural experiment of regionalization in California to determine the extent to which disparities in access, treatment, and outcomes have changed for patients with STEMI who are residents in minority communities in regionalized vs nonregionalized counties. This study hypothesizes that residents of communities with high proportions of racial/ethnic minorities may not experience the same improvements in access, treatment, and outcomes postregionalization relative to residents in nonminority communities who experience STEMI.

## Methods

Our study follows the Strengthening the Reporting of Observational Studies in Epidemiology (STROBE) reporting guideline. Institutional review board approval for this study was provided by the University of California–San Francisco. Informed consent was not required because deidentified administrative databases were used for this study.

### Population-Based Approach

Because randomized clinical trials are infeasible for these types of policy interventions, the methodological challenge to studying effects of STEMI regionalization policies is ensuring that any changes found during implementation are solely due to the policy itself, rather than any other changes that might have occurred during this time. The conventional approach at the individual level using a simple difference model inappropriately attributes all changes to the policy itself and does not evaluate the effect on patients in a regionalized network who are not admitted to a PCI hospital. Given that regionalization policies affect patients with STEMI who are admitted to hospitals with and without PCI, a community perspective using a comparison group that did not experience regionalization is more desirable because it allows a population-based approach to evaluate and appropriately attribute any benefits to the regionalization policy. This is particularly important because many patients with STEMI do not arrive via ambulance and placement of STEMI hospitals might be such that some patients would face a trade-off between further travel time and a hospital capable of cardiovascular interventions.

### Definition of Minority Community

This analysis focused on racial minorities who are known to have access barriers and disparities at the community level (as opposed to the individual level). A zip code community is defined as a minority community if its share of the Black or Hispanic population is at the top tertile of the overall California distribution, based on 2000 Census data. According to this definition, 47% of the patients with STEMI in this study resided in minority communities (among them, 46% were White, 10% were Black, and 28% were Hispanic), and 53% resided in nonminority communities (among them, 77% were White, 2% Black, and 8% Hispanic) (Table 1).

**Table 1.** Characteristics of Patients, Hospitals, and Communities in This Study

Characteristic	No. (%)		
	All patients	Communities	
		Nonminority	Minority
**Patient**			
Women	45 800 (32.8)	24 072 (32.8)	21 728 (32.9)
Race/ethnicity			
White (non-Hispanic)	86 392 (61.9)	56 218 (76.5)	30 174 (45.7)
Black	7804 (5.6)	1187 (1.6)	6617 (10.0)
Hispanic	24 778 (17.8)	6146 (8.4)	18 632 (28.2)
Asian	12 620 (9.0)	5820 (7.9)	6800 (10.3)
Other/mixed	7900 (5.7)	4074 (5.5)	3826 (5.8)
Age, y			
40-64	63 959 (45.9)	31 607 (43.0)	32 352 (49.0)
65-69	16 419 (11.8)	8623 (11.7)	7796 (11.8)
70-74	13 831 (9.9)	7426 (10.1)	6405 (9.7)
75-79	12 862 (9.2)	7061 (9.6)	5801 (8.8)
80-84	12 423 (8.9)	7191 (9.8)	5232 (7.9)
85-99	16 459 (11.8)	9979 (13.6)	6480 (9.8)
Insurance (expected source of payment)			
Private	44 058 (31.6)	24 277 (33.1)	19 781 (29.9)
Medicare	67 898 (48.7)	37 861 (51.6)	30 037 (45.5)
Medicaid	13 230 (9.5)	4897 (6.7)	8333 (12.6)
Indigent (county or other)	3913 (2.8)	1714 (2.3)	2199 (3.3)
Self-pay	7243 (5.2)	3136 (4.3)	4107 (6.2)
Other	3152 (2.3)	1560 (2.1)	1592 (2.4)
Patient comorbid conditions			
Peripheral vascular disease	12 604 (9.0)	6576 (9.0)	6028 (9.1)
Pulmonary circulation disorders	3655 (2.6)	2000 (2.7)	1655 (2.5)
Diabetes	44 276 (31.7)	19 686 (26.8)	24 590 (37.2)
Kidney failure	19 496 (14.0)	9454 (12.9)	10 042 (15.2)
Liver disease	2094 (1.5)	985 (1.3)	1109 (1.7)
Cancer	3706 (2.7)	2054 (2.8)	1652 (2.5)
Dementia	2826 (2.0)	1568 (2.1)	1258 (1.9)
Valvular disease	11 855 (8.5)	6960 (9.5)	4895 (7.4)
Hypertension	93 539 (67.1)	47 606 (64.8)	45 933 (69.5)
Chronic pulmonary disease	21 363 (15.3)	11 273 (15.3)	10 090 (15.3)
Rheumatoid arthritis/collagen vascular	2580 (1.8)	1492 (2.0)	1088 (1.6)
Coagulation deficiency	6077 (4.4)	3110 (4.2)	2967 (4.5)
Obesity	17 956 (12.9)	9029 (12.3)	8927 (13.5)
Substance abuse	8200 (5.9)	3726 (5.1)	4474 (6.8)
Depression	6550 (4.7)	3692 (5.0)	2858 (4.3)
Psychosis	3234 (2.3)	1550 (2.1)	1684 (2.5)
Hypothyroidism	11 596 (8.3)	6888 (9.4)	4708 (7.1)
Paralysis and other neurologic disorder	9599 (6.9)	4963 (6.8)	4636 (7.0)
Chronic peptic ulcer disease	58	31	27
Weight loss	3111 (2.2)	1484 (2.0)	1627 (2.5)
Fluid and electrolyte disorders	24 421 (17.5)	12 592 (17.1)	11 829 (17.9)
Anemia	20 764 (14.9)	10 320 (14.1)	10 444 (15.8)
**Admitting hospital **			
Ownership			
For profit	20 787 (14.9)	10 990 (15.0)	9797 (14.8)
Government	17 386 (12.5)	7660 (10.4)	9726 (14.7)
Teaching hospital^a^	13 941 (10.0)	5704 (7.8)	8237 (12.5)
Hospital is part of a system	88 624 (63.5)	48 208 (65.6)	40 416 (61.2)
No. of beds, mean (SD)	403.03 (4633.41)	379.57 (4294.9)	429.08 (4982.22)
Occupancy rate, mean (SD)^b^	0.66 (0.14)	0.66 (0.14)	0.67 (0.14)
HHI within 15 miles based on total discharges, mean (SD)	0.23 (0.24)	0.25 (0.25)	0.2 (0.23)
CABG availability	103 655 (74.3)	54 811 (74.6)	48 844 (74.0)
PCI laboratory availability	112 326 (80.5)	59 837 (81.5)	52 489 (79.5)
Community financial characteristics			
County population, mean (SD)	3 572 470.03 (3 772 053.8)	2 822 434.2 (3 344 752.71)	4 406 492.96 (4 035 962.58)
Per capita income, mean (SD), $	43 061.85 (11 700.47)	44 987.09 (12 597.85)	40 919.21 (10 194.12)
Live in low-income zip code communities (lowest quartile of family income distribution)	33 102 (23.7)	15 244 (20.8)	17 858 (27.0)
No. of observations	139 494	73 445	66 049

Abbreviations: CABG, coronary artery bypass graft; HHI, Herfindahl-Hirschman Index; PCI, percutaneous coronary intervention.

^a^ If resident-to-bed ratio greater than 0.25.

^b^ Total inpatient days/available beds.

We used geographic boundaries to define communities because geographic variation in health care resources, such as PCI availability, is highly correlated with health outcomes.^44,45^ All residents (regardless of individual race and ethnicity) who resided in the same communities had the same value on their minority community indicator, but we controlled for individual patients’ race categories in our statistical models. We used the crosswalk data provided by the US Department of Housing and Urban Development's Office of Policy Development and Research to map zip code communities into counties.^46^

### Data Sources

Several databases were linked to perform this study. First, nonpublic inpatient data between January 1, 2006, and October 31, 2015, from the California Office of Statewide Health Planning and Development were used, containing patients’ zip codes, admission dates, source of admission, demographics (eg, age, sex, race/ethnic groups), insurance status at admission, *International Classification of Diseases, Ninth Revision (ICD-9)* diagnostic codes, treatments received (identified through 21 *ICD-9* procedure codes, as well as their dates), comorbidities, disposition, and date of death. Second, these data were linked with Office of Statewide Health Planning and Development nonpublic emergency department data from 2006 to 2015, using a unique patient identifier, and also merged with vital statistics data, allowing capture of a complete patient cohort. Third, detailed regionalized care arrangement information was collected from all 33 local EMS agencies, representing all 58 counties in the state (because not all small, more rural, and less populated counties have their own EMS agency) through survey.^47,48^ This data set identifies dates of implementation of STEMI regionalization and details of these protocols from each local EMS agency. Fourth, zip-code-level population characteristics from the 2010 US Census were extracted to identify communities that have baseline high levels of Black and Hispanic population. Fifth, with hospital identifier on the discharge data, facility data were merged to capture hospital characteristics from several sources: the American Hospital Association annual surveys (ownership, system membership, and number of hospital beds), the Healthcare Cost Reporting Information System from the Centers for Medicare & Medicaid Services (teaching status, case mix index, occupancy rate, and total discharges), and Office of Statewide Health Planning and Development facility use data (total facility procedure volume and emergency department availability).

### Definition of Regionalization

Although regionalization is used broadly in the literature to connote the idea of “matching of medical resources to patient needs to maximize health benefits and outcomes while minimizing cost and use of resources over a specified geographic area,”^49^ regionalized STEMI networks necessitate complex organization across various sectors of the health care system, including agreement by the majority of health systems, hospitals, physician groups, and EMS agencies within a region to work in a coordinated fashion using common protocols, as well as provide ongoing measurement and feedback regularly. Theoretically, regionalization of services in an area could entail a hospital’s voluntarily closing their services. Regionalization in the United States, however, remains within the context of a largely privatized hospital system governed by financial incentives. In fact, in the United States, regionalization has been accompanied by a secular trend in an increase in hospitals offering PCI^50^ who may not want to be deprived of potential revenue because smaller community hospitals have feared losing the patient volume associated with cardiovascular care that would be potentially diverted owing to regionalization.^51^ Given that cardiovascular services are estimated to account for 35% or more of a community’s hospital revenue,^52^ if PCI hospitals are preferentially located in wealthier areas,^34,35^ community hospitals in poorer areas that could have a higher proportion of minorities could be differentially affected. These realities provide the basis for analyzing patients by geographic community of residence.

We designed a survey (described fully elsewhere^53,54^) that objectively categorized a system’s regionalization based on class I recommendations from the American College of Cardiology and American Heart Association categorization requiring that a STEMI regionalized network have an emergency medical system that instructs prehospital transport to directly transport patients with STEMI to facilities that offer emergency PCI, bypassing hospitals that do not offer it, and have interhospital transfer protocols specifically for patients with STEMI.^25^ This survey was deployed across all local EMS agencies in California and achieved a 100% response rate. Counties were initially categorized into various levels of regionalization (none, partial, and substantial). After models were run showing that there were no significant differences based on these more granular categorizations and that the effects of regionalization were almost all concentrated when a county changed from “none” to “partial” and that there was little difference between changes from “partial” to “substantial,” we chose a more parsimonious main model that combined the partial and substantial regionalization into 1 “regionalized” category.

### Patient Cohort

We identified patients with STEMI as those whose primary diagnosis was 410.x0 or 410.x1 according to *ICD-9-CM* (eMethods in the Supplement contains descriptions), excluding 410.7x, using a previously validated approach.^2,55^

### Patient Outcomes

Three outcomes were analyzed in this study: access to PCI-capable hospitals, treatment, and mortality. First, access to PCI-capable hospitals was defined by admission to a hospital with PCI capability, using a volume threshold from prior literature in which a hospital is PCI capable if its annual volume is 50 or more.^50^ Second, treatment was defined as receipt of PCI on the same day and at any time during the hospitalization. Because the patient cohort consisted of those receiving a diagnosis of STEMI, coronary angiography was included in our definition of PCI to capture attempts at intervention. While PCI is generally the definitive treatment for STEMI, inclusion of coronary angiography accounts for clinical realities of failed PCI attempts, false-positive diagnoses of STEMI, and referral to coronary artery bypass graft in cases in which such intervention would be clinically preferred over PCI. The third set of outcomes was a direct assessment of patient health: time-specific all-cause mortality at 30, 90, or 365 days.

### Statistical Analysis

The unit of analysis was the patient. One key strength of this analysis was the use of longitudinal data that allowed us to track pre-post changes in access, treatment, and health outcomes between patients in minority and nonminority communities when both were exposed to a STEMI regionalized network. We used a difference-in-difference-in-differences estimation approach that incorporated county fixed effects. Under the difference-in-difference-in-differences framework, the main model compared changes in PCI access, PCI received, and health outcomes between minority and nonminority communities before and after they became part of a STEMI regionalized network.

Although all outcomes were dichotomous, a linear probability model with county fixed effects was used (for more discussion regarding choice of model, see eMethods in the Supplement). There were 2 key components to the main model. First, the overall STEMI network effect on the dependent variable was identified through an indicator that took on the value of 1 on and after the year that a patient’s community switched to a STEMI regionalized network. Second, differential changes in outcomes for patients between minority and nonminority communities when both were exposed to a regionalized network were distinguished by including an interaction term between a community’s minority status and STEMI network indicator. In addition, an indicator for minority community was included to capture baseline differences between minority and nonminority communities. Third, county fixed effects and time dummies were included to remove unobserved underlying differences in patient population and practice pattern across counties, as well as to account for secular trend in dependent variables that were common across all communities.

All models controlled for a patient’s individual race and ethnicity (White, Black, Hispanic, Asian, and other), insurance categories (private, Medicare, Medicaid, indigent care, self-pay, and other), other patient demographic covariates (5-year age groups and sex), whether a patient was a transfer from another hospital, as well as 22 Elixhauser patient comorbid indicators to control for underlying individual patient health conditions.^41,55^ In essence, the aforementioned model asked the following hypothetical question: did 2 patients who were comparable in all observed dimensions derive similar benefits from a STEMI network if one resided in a minority community and the other resided in a nonminority community?

Our main model focused on capturing overall differences between minority and nonminority communities when both were exposed to regionalization. In our second model, we further explored potential differences between minority and White patients across minority and nonminority communities when exposed to regionalization. We replaced the 2 key components from the main model with the following set of indicators that turned to 1 when and after each of the following categories of patients was exposed to regionalization: White patients in nonminority communities, Black or Hispanic patients in nonminority communities, White patients in minority communities, and Black or Hispanic patients in minority communities. The rest of the independent variables were identical to those in the main model. The sensitivity analyses are described in the Results section. The threshold for statistical significance was set at 2-tailed *P* < .05. We performed all analyses with Stata version 15.

## Results

**Figure 1.**
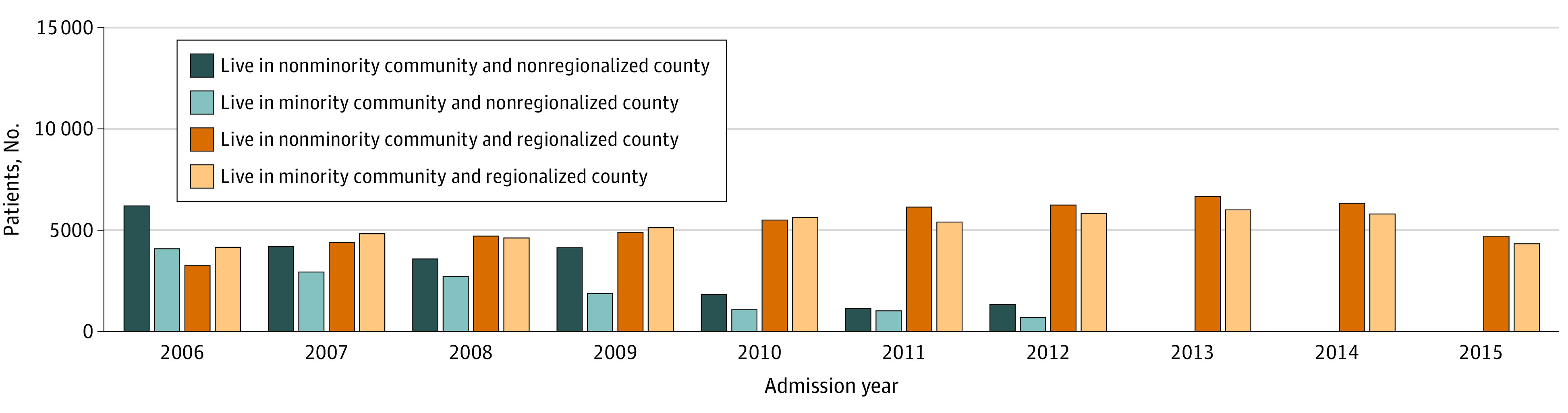
Proportion of Patients by Community Minority Status and County Regionalization Status, 2006-2015

This study included 139 494 patients with STEMI; 61.9% of patients were non-Hispanic White, 5.6% Black, 17.8% Hispanic, and 9.0% Asian; 32.8% were women. Table 1 shows the demographic and other characteristics of the study population overall and stratified by nonminority (n = 73 445) and minority (n = 66 049) communities. Figure 1 contains the proportion of patients by community minority status and county regionalization status during the course of our study, illustrating the gradual decline (and disappearance) of nonregionalized counties by 2013. Within nonminority communities, 76.5% were non-Hispanic White patients, 1.6% Black, 8.4% Hispanic, 7.9% Asian, and 5.5% other races. Within minority communities, 45.7% were non-Hispanic White, 10.0% Black, 28.2% Hispanic, 10.3% Asian, and 5.8% other races. Patients in nonminority communities were slightly older, on the whole, compared with those in minority communities, with a higher proportion of private and Medicare insurance coverage. Patients living in minority communities were more likely to have Medicaid (12.6% vs 6.7%), and had higher rates of diabetes (37.2% vs 26.8%), kidney failure (15.2% vs 12.9%), and hypertension (69.5% vs 64.8%). Residents of minority communities also tended to be admitted to a higher proportion of government-owned and teaching hospitals with greater bed size, and in areas with a lower Herfindahl-Hirschman Index, indicating more hospital competition. Minority communities additionally tended to be more populated and have a lower mean per capita income ($40 919 vs $44 987).

In our main model (Table 2) (full results reported in eTable 1 in the Supplement), accounting for individual patient race/ethnicity and other patient and hospital-level characteristics, we found improvement in access to PCI-capable hospitals by 6.3 percentage points (95% CI, 5.5 to 7.1 percentage points; *P* < .001) for residents in nonminority communities after they were exposed to regionalization. This is equivalent to an 8.7% improvement in access (from the baseline mean of 72.7%). Patients in a minority community had a smaller improvement in PCI access, by 1.8 percentage points (95% CI, −2.8 to −0.8 percentage points; *P* < .001) compared with residents in nonminority communities when both became regionalized. Similarly, when treatments were evaluated, the improvements from regionalization in the receipt of same-day PCI and PCI at any time during the hospitalization were also smaller for patients in minority communities compared with nonminority communities. Specifically, for same-day or PCI at any point in the hospitalization for residents of nonminority communities, regionalization was associated with an increase of 5.1 percentage points (95% CI, 4.2 to 6.1 percentage points; *P* < .001) and 5.0 percentage points (95% CI, 4.2 to 5.9 percentage points; *P* < .001), respectively. In comparing patients in nonminority vs minority communities, improvement in PCI treatment was reduced by 3.4 percentage points (95% CI, −4.5 to −2.2 percentage points; *P* < .001) for same-day and by 4.3 percentage points for PCI during the hospitalization (95% CI, −5.3 to −3.2 percentage points; *P* < .001) for patients in minority communities when both were exposed to regionalization. In other words, the improvement experienced by patients in minority communities as the result of regionalization represented only 33.3% and 15.1% of the benefit experienced by patients in nonminority communities. For easier comparison, the probability of patients’ receiving same-day PCI in nonminority communities because of regionalization increased by 10.3% (5.1 percentage points off 49.7% baseline) after regionalization, whereas the probability of receiving PCI on the same day of admission for patients in minority communities increased by only 3.4% (5.1−3.4; 1.7 percentage points off the 49.7% baseline).

**Table 2.** Regression-Adjusted Percentage Point Changes in Outcomes Between Nonminority and Minority Communities After Both Were Exposed to Regionalization

	PCI			Mortality		
	Admitted to hospital	Received on same day	Received during the episode	30 d	90 d	1 y
No.	135 579	139 257	139 257	117 896	117 896	117 896
Sample mean at baseline, %	72.7	49.7	64.2	13.6	16.6	21.4
Baseline differences between nonminority and minority communities (95% CI)	1.5 (0.5 to 2.4)	0.6 (−0.4 to 1.7)	2.2 (1.3 to 3.2)	0.8 (0.0 to 1.5)	0.9 (0.1 to 1.7)	0.9 (0.0 to 1.7)
Changes in outcome after regionalization of nonminority county (95% CI)	6.3 (5.5 to 7.1)	5.1 (4.2 to 6.1)	5.0 (4.2 to 5.9)	−0.5 (−1.3 to 0.2)	−0.6 (−1.3 to 0.2)	−0.6 (−1.4 to 0.2)
Additional change in outcome in minority communities relative to nonminority (95% CI)	−1.8 (−2.8 to −0.8)	−3.4 (−4.5 to −2.2)	−4.3 (−5.3 to −3.2)	0.2 (−0.6 to 1.0)	0.4 (−0.5 to 1.3)	0.7 (−0.2 to 1.6)

Abbreviation: PCI, percutaneous coronary intervention.

**Figure 2.**
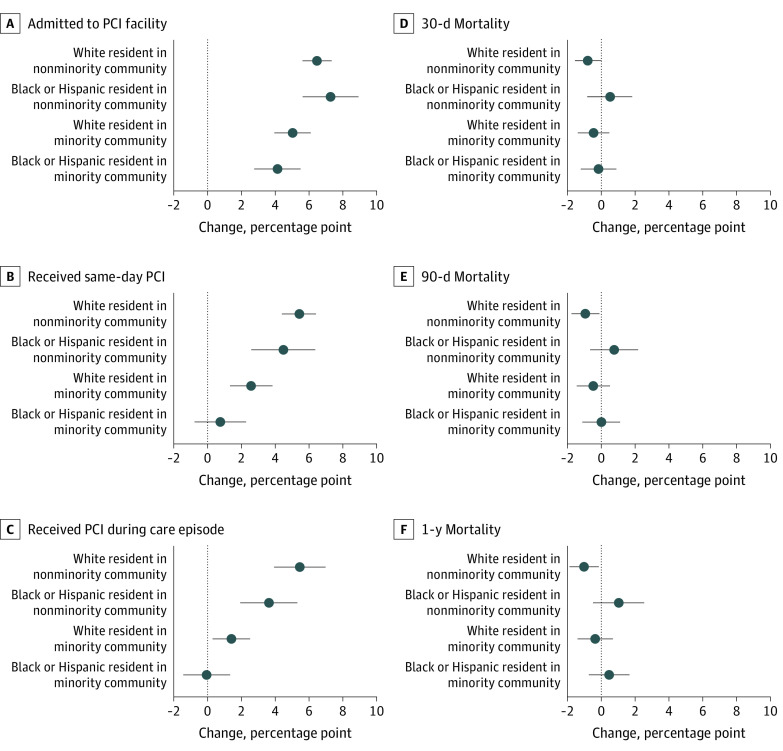
Regression-Adjusted Percentage Point Changes in Outcomes by Minority Status at Individual and Community Levels After Exposure to Regionalization Abbreviation: PCI, percutaneous coronary intervention.

Figure 2 illustrates changes in outcome by individual and community-level designations of minority status after each group was exposed to regionalization, based on model 2 results (full regression results are included in eTable 2 in the Supplement). The left panel of Figure 2 shows a clear trend of the largest disparities between the benefits that White patients in nonminority communities received compared with Black or Hispanic patients in minority communities. After regionalization, White individuals in nonminority communities experienced a 5.4 percentage point increase (95% CI, 4.4 to 6.4 percentage points; *P* < .001) in the probability of receiving same-day PCI, but Black or Hispanic patients with STEMI who were living in minority communities did not accrue any benefit (0.8 percentage points; 95% CI, −0.8 to 2.3 percentage points; *P* = .33). Similarly, the probability of receiving PCI during the hospitalization for White patients in nonminority communities after regionalization increased by 5.5 percentage points (95% CI, 4.6 to 6.4 percentage points; *P* < .001), but not for Black or Hispanic patients in minority communities (−0.1 percentage points; 95% CI, −1.5 to 1.3 percentage points; *P* = .93). For mortality, White residents of nonminority communities experienced improvements in 30-day, 90-day, and 1-year mortality (percentage point change: −0.8, 95% CI, −1.6 to 0, *P* = .05; −0.9, 95% CI, −1.8 to −0.1, *P* = .03; and −1.0, 95% CI, −1.9 to −0.2, *P* = .02, respectively). None of these improvements in mortality were experienced by other groups; specifically, White residents of minority communities and Black or Hispanic residents of nonminority and minority communities.

We performed 5 additional sensitivity analyses and included their results in the Supplement: restricting our patient population, using propensity scores to match counties with similar preregionalization mortality trends (eTable 3 in the Supplement); controlling for PCI access to investigate whether the differentials we observed between minority and nonminority communities in treatment and outcomes were driven by PCI access (eTable 4 and eFigure in the Supplement); excluding patients with STEMI whose principal diagnostic code was *ICD-9-CM* code 410.9 because there is some literature^55^ that suggests this could be more non-STEMI than STEMI (eTable 5 in the Supplement); using a narrower definition of PCI that excludes coronary angiography (eTable 6 in the Supplement); and using a narrower definition of minority community, with minority defined as zip codes whose Black or Hispanic share of the population was in the top quartile instead of tertile of the distribution (eTable 7 in the Supplement). Our results were robust across all sensitivity analyses and our conclusions remained the same. In addition, the second sensitivity analysis revealed that the main mechanism through which regionalization improved patient care was through improved access to PCI –capable hospitals.

## Discussion

We found that patients in minority communities with STEMI derived smaller benefits from cardiac care regionalization than those in nonminority communities. Specifically, compared with patients in nonminority communities, those with STEMI in minority communities benefited less in the likelihood of being admitted to a PCI-capable hospital and actual treatment as measured by receipt of PCI, both on the same day and at any point in the hospitalization. When community-level minority designation was examined, neither group received any benefit when mortality was measured from regionalization, consistent with the null effect on mortality from other literature using comparable populations before and after regionalization.^1,2,39,56^ However, our additional analysis showed that White patients in nonminority communities experienced a mortality improvement (equating to a 5.9% decrease in 30-day mortality, for example) when exposed to regionalization, but other groups (White patients in minority communities and Black or Hispanic patients in either type of community) had little or no improvement when exposed to regionalization.

What can explain this unsettling finding of differential benefit for patients in minority communities? There are 2 potential explanations. First, prehospital factors could be an explanation for decreased benefit for patients in minority communities because of systematic sorting (or preferences) of patients into different hospitals. Patients with STEMI who use EMS transport are more likely to be taken to PCI-capable hospitals initially compared with those who arrive at hospitals by self-transport, and even for patients with STEMI, EMS is used only 60% of the time.^57^ It is possible that patients with STEMI from minority communities use EMS less often than those from nonminority communities, as observed in data from stroke patients,^58,59^ or that patients with STEMI in minority communities have longer transport times.^60^

Perhaps a larger contributor to these inequities in access, treatment, and outcome, however, is that practice patterns in hospitals or regions that serve patients in minority communities are systemically different from those that serve nonminority communities. Existing literature suggests that intrahospital variation in treatment of patients may be a very small component compared with interhospital variation.^61^ Comparison of the results from our main analysis and the sensitivity analysis (eTable 4 and eFigure in the Supplement, where regionalization’s effect on receipt of same-day and in-hospital PCI is greatly reduced when access to a PCI-capable hospital is taken into account) demonstrates that one mechanism through which regionalization appears to have increased PCI treatment and improved outcomes is through improved access, or admission, to a PCI hospital. In other words, the organized routing and presence of transfer protocols inherent in regionalization for critical care patients to some extent appears to have mitigated factors previously cited that keep patients away from high-volume hospitals, such as knowledge of options, provider referral preferences, and patient preferences,^62^ that may keep underserved patient populations in a non-PCI hospital during a STEMI care situation.

However, whether a patient lives in a nonminority or minority community still plays a sizeable role in determining the extent of the potential benefit of regionalization even after the model controls for access to a PCI-capable hospital. Current evidence regarding this type of structural racism^63^—in which the opportunities for health differ by race because of mutually reinforcing systemic inequities in society^64^—indicates that this may indeed be the case, in which hospitals serving high proportions of minority patients tend to be of lower quality,^65,66^ both in cardiac care^7,66,67,68^ and in other conditions such as surgery.^69^ One underlying mechanism explaining this phenomenon could be fewer resources and decreased ability to perform certain procedures in hospitals that serve patients from minority communities. Given that both emergency care in general and PCI specifically are less available in underserved communities,^34,45,70^ PCI hospitals in minority communities could already be burdened by a high volume of patients as the result of regionalization and less able to provide guideline-directed care. Simply directing more patients with STEMI to those facilities may not result in an increase in the proportion of patients receiving PCI, as observed in a cohort of Veterans Affairs patients requiring angiography and being treated in the Veterans Affairs health care system.^71^

The gradation of benefit at the intersection of both the individual and community-level minority designations as shown in Figure 2 supports the idea that there are complex and powerful structures influencing issues of access, treatment, and outcomes, with the most benefit accruing to White patients living in nonminority communities, followed by Black or Hispanic patients living in nonminority communities, then White patients living in minority communities, and last, Black or Hispanic patients living in minority communities. Even when access to a PCI hospital is controlled for in the models (eFigure in the Supplement), this gradation remains.

These findings highlight the importance of incorporating the community-level approach to evaluate disparities. To our knowledge, there are no population-level studies of regionalization in the United States with a similar control group, despite the calls for formal evaluation using population-based data for STEMI programs. The only study so far with any control group used 2 analyses, one at the hospital level within North Carolina that was fairly limited in power and another using Medicare patients in North Carolina compared with Medicare patients nationally, which was not representative of the patients receiving the intervention.^2^ This is a significant deficiency in perspective, given that regionalization is typically implemented in an effort to better treat the entire community, which hospital-cohort studies are unable to evaluate. In fact, analysis of our data using the individual perspective alone without the community yielded the finding that regionalization did not affect minority patients differentially. Our community analysis allowed us to transcend the averages: that patients in minority communities do not experience the same benefits from regionalization as those in nonminority communities, and that there are further disparities at the individual level of race even within the same communities. It is also possible that these types of analyses are increasingly important when certain segments of the population may be more invested in the results when they realize potential effects of disparities on their own health; in our sample, there were a nontrivial number (45.7%) of White patients living in nonminority communities.

Unlike that of other observational studies, our study design allowed us to exclude the possibility that these were due to secular improvements in care^55,72^ or regional variation and more definitively observe the effects of regionalization directly.^61^ A national study comparing 6 states with emergency department bypass policies with 6 similar states without bypass policies in a Mission: Lifeline cohort determined that patients with STEMI from states with bypass policies were more likely to be White.^48^ Our findings showed that the effects of regionalization on increasing disparities are not only due to such systemic disparities such as where bypass policies exist, or even due to the location of designated PCI centers, which tend to be located in affluent communities,^34^ but also that the benefits of technologic advances such as those in cardiac care do not accrue to disadvantaged populations, likely because of existing structural barriers and biases in the health care system. A crucial implication of this research is that populationwide initiatives must be implemented with careful thought about how technology may not be an equalizer, as previously thought, but may in fact widen disparities.

### Limitations

Our analysis has limitations. First, although the general framework for implementing STEMI regionalized care is well established, policies can vary considerably across states. We evaluated only the STEMI regionalization movement in California, and although California represents 12% of the US population, our results may not be generalizable to the rest of the United States, especially not to rural areas. Second, although fixed effects remove time-invariant unobserved differences across counties under different regionalization arrangement, our results might be biased because of unobserved time-varying characteristics associated with regionalization and health outcomes not captured in the data. The results in our first sensitivity analysis (eTable 1 in the Supplement) using a subset of observations in which the counties had similar mortality trend in the preregionalization period (ie, between 2001 and 2005) remained the same. Third, we relied on patient discharge data, which have limited clinical information. However, more detailed data that contain richer clinical information, such as the CathPCI Registry, capture only patients who received PCI, and therefore preclude evaluation of all patients with STEMI in an integrated STEMI system. Similar registries, such as the American College of Cardiology/American Heart Association’s ACTION Registry–Get With the Guidelines, which have been so valuable for understanding specific treatments and precise timing intervals in the cardiovascular literature, are of limited use in our population-based study because the majority of hospitals in these registries are STEMI receiving hospitals. The marked absence of STEMI referral hospitals would render us unable to determine differences among communities, and the registries also do not capture time-specific mortality measures, which are 2 distinctive goals in our study. This is one reason that the percentage of patients with STEMI receiving PCI in our population-based study, which comprised all hospitals, including the small hospitals not capable of PCI, may be lower than that in the majority of published studies that rely on registry data to provide estimates of STEMI treatment for PCI patients. Fourth, although we were able to document the differential benefits of regionalization for patients between minority and nonminority communities and explore some mechanisms, we could not fully explore all possible mechanisms that might explain the differentials. For example, we were unable to capture whether ambulance use differs between these 2 types of communities, nor were we able to capture whether bypass policies systematically differ between minority and nonminority communities. To reduce disparity when these local initiatives are implemented, it would be important to delve deeper into the nuances of local policies and care-seeking patterns.

## Conclusions

Our findings contribute to the national conversation on ever-widening health disparities and structural barriers in the United States by suggesting that we may not be able to rely solely on generalized initiatives such as regionalization to close gaps between minority and nonminority individuals, and minority and nonminority communities. Our findings also highlight the importance of examining the effects of systemwide regionalization initiatives by subgroups because analyses that are limited to outcomes on the average population may miss important trends in certain underrepresented communities. Finally, analyzing outcomes by subgroups may also reveal important benefits of interventions—in this case, a mortality benefit—that would be (and has been) otherwise undetected. These advances in knowledge allow a deeper understanding of the mechanisms of these interventions to enable better targeting and refinement of future health care programs, especially regarding population-based interventions for patients in vulnerable communities.

## Notes

Supplement. **eMethods.** Technical Appendix **eTable 1.** Full regression results for Table 2 **eTable 2.** Full regression results for Figure 2 **eTable 3.** Sensitivity analysis using propensity score methods to match counties with similar pre-regionalization mortality trend **eTable 4.** Sensitivity analysis of main model controlling for admission to PCI hospital **eTable 5.** Sensitivity analysis excluding 410.9x **eTable 6.** Sensitivity analysis using alternative definition of PCI **eTable 7.** Sensitivity analysis using alternative definition of minority community **eFigure.** Regression-adjusted percentage point changes in outcomes by minority status at individual and community levels after exposure to regionalization when controlling for access to PCI capable hospitals
