# Supplementary material for: Evaluation of STEMI Regionalization on Access, Treatment, and Outcomes Among Adults Living in Nonminority and Minority Communities
Source: JAMA Netw Open. 2020 Nov 16;3(11):e2025874. doi: 10.1001/jamanetworkopen.2020.25874 (PMC7670311; doi:10.1001/jamanetworkopen.2020.25874)
Supplement: Supplement. — eMethods. Technical Appendix eTable 1. Full regression results for Table 2 eTable 2. Full regression results for Figure 2 eTable 3. Sensitivity analysis using propensity score methods to match counties with similar pre-regionalization mortality trend eTable 4. Sensitivity analysis of main model controlling for admission to PCI hospital eTable 5. Sensitivity analysis excluding 410.9x eTable 6. Sensitivity analysis using alternative definition of PCI eTable 7. Sensitivity analysis using alternative definition of minority community eFigure. Regression-adjusted percentage point changes in outcomes by minority status at individual and community levels after exposure to regionalization when controlling for access to PCI capable hospitals [file jamanetwopen-e2025874-s001.pdf]

Supplemental Online Content

Hsia RY, Krumholz H, Shen YC. Evaluation of STEMI regionalization on access, treatment, and outcomes among adults living in nonminority and minority communities. *JAMA Network Open*. 2020;3(11):e2025874. doi:10.1001/jamanetworkopen.2020.25874

eMethods: Technical Appendix
eTable 1. Full regression results for Table 2
eTable 2. Full regression results for Figure 2
eTable 3. Sensitivity analysis using propensity score methods to match counties with similar pre-regionalization mortality trend
eTable 4. Sensitivity analysis of main model controlling for admission to PCI hospital
eTable 5. Sensitivity analysis excluding 410.9x
eTable 6. Sensitivity analysis using alternative definition of PCI
eTable 7. Sensitivity analysis using alternative definition of minority community
eFigure. Regression-adjusted percentage point changes in outcomes by minority status at individual and community levels after exposure to regionalization when controlling for access to PCI capable hospitals

This supplemental material has been provided by the authors to give readers additional information about their work.

© 2020 Hsia et al. *JAMA Network Open.*

eMethods: Technical Appendix

Description of ICD-9-CM codes used in study
410.00 Acute myocardial infarction of anterolateral wall, episode of care unspecified
410.01 Acute myocardial infarction of anterolateral wall, initial episode of care
410.10 Acute myocardial infarction of other anterior wall, episode of care unspecified
410.11 Acute myocardial infarction of other anterior wall, initial episode of care
410.20 Acute myocardial infarction of inferolateral wall, episode of care unspecified
410.21 Acute myocardial infarction of inferolateral wall, initial episode of care
410.30 Acute myocardial infarction of inferoposterior wall, episode of care unspecified
410.31 Acute myocardial infarction of inferoposterior wall, initial episode of care
410.40 Acute myocardial infarction of other inferior wall, episode of care unspecified
410.41 Acute myocardial infarction of other inferior wall, initial episode of care
410.50 Acute myocardial infarction of other lateral wall, episode of care unspecified
410.51 Acute myocardial infarction of other lateral wall, initial episode of care
410.60 True posterior wall infarction, episode of care unspecified
410.61 True posterior wall infarction, initial episode of care
410.70 Subendocardial infarction, episode of care unspecified
410.71 Subendocardial infarction, initial episode of care
410.80 Acute myocardial infarction of other specified sites, episode of care unspecified
410.81 Acute myocardial infarction of other specified sites, initial episode of care
410.90 Acute myocardial infarction of unspecified site, episode of care unspecified
410.91 Acute myocardial infarction of unspecified site, initial episode of care

Selection of Linear Probability Model v. Probit or Logit Model
Even though a probit or logit model is a natural choice for estimating a dichotomous variable in cross-sectional data, these models result in inconsistent estimators in panel data because we are including a large number of fixed effects[1] The linear probability models can consistently estimate the effect of STEMI on dichotomous outcomes.[2] One drawback of the model is that the predicted probability can be out of bounds. In our prior work analyzing a dichotomous outcome for AMI patients, we obtained virtually no out-of-bound predictions among the 1.49 million observations in our analysis. Another concern is that error term is heteroskedastic. We correct this problem by estimating heteroskedasticity-robust standard errors that include adjustment for clustering within counties, and such an estimate is consistent in the fixed-effects.[3]
Another important benefit of using a linear probability model in our context is because our key variable is an interaction term, and using a linear probability model allows us to readily convey the magnitude of the effect to the readers while avoiding the incidental parameter problem. We cannot easily interpret the odds ratio of an interaction term directly from a logit model except for the direction of the association and statistical significance.
As a robustness check, we did estimate a logit regression that include the 56 county dummies. Our conclusions remain the same. Given the interpretation and the incidental parameter issue, we felt that using linear probability with county fixed effects is still the most appropriate model in this context.
Lastly, for our purposes, the fixed-effects model is superior over the hierarchical model in that it removes unobserved time-invariant differences across counties. Hierarchical models, on the other hand, model between-county variability as a random effect, and therefore cannot remove influences of unobserved differences across counties, such as care-seeking behaviors of the underlying population and baseline condition that might correlate with regionalization. The ability to remove unobserved differences across counties is critical.

eReferences

1. Greene WH. *Econometric Analysis*. 7th ed. Prentice Hall; 2012.
2. Buchmueller TC, Jacobson M, Wold C. How far to the hospital?: The effect of hospital closures on access to care. *J Health Econ*. 2006;25(4):740-761. doi:10.1016/j.jhealeco.2005.10.006
3. Stock JH, Watson MW. Heteroskedasticity-Robust Standard Errors for Fixed Effects Panel Data Regression. *Econometrica*. 2008;76(1):155-174. doi:10.1111/j.0012-9682.2008.00821.x

© 2020 Hsia et al. *JAMA Network Open.*

eTable 1. Full regression results for Table 2

Coefficient [95% CI]	**Admitted to PCI hospital**	**Receive PCI on the same day**	**Receive PCI during the episode**	**30-day mortality**	**90-day mortality**	**1-year mortality**
**Exposure variables**						
Baseline differences b/w non-minority and minority	1.5**	0.6	2.2**	0.8*	0.9*	0.9*
communities	[0.5,2.4]	[-0.4,1.7]	[1.3,3.2]	[0.0,1.5]	[0.1,1.7]	[0.0,1.7]
Changes in outcome after non-minority county is	6.3**	5.1**	5.0**	-0.5	-0.6	-0.6
regionalized	[5.5,7.1]	[4.2,6.1]	[4.2,5.9]	[-1.3,0.2]	[-1.3,0.2]	[-1.4,0.2]
Additional change in outcome in minority communities relative to non-minority	-1.8**	-3.4**	-4.3**	0.2	0.4	0.7
	[-2.8,-0.8]	[-4.5,-2.2]	[-5.3,-3.2]	[-0.6,1.0]	[-0.5,1.3]	[-0.2,1.6]
**Patient characteristics**						
Race (reference: White)						
African American	-4.6**	-7.1**	-7.5**	-0.9*	-1.1*	0.3
	[-5.6,-3.7]	[-8.2,-6.0]	[-8.5,-6.4]	[-1.7,-0.1]	[-1.9,-0.2]	[-0.6,1.3]
Hispanic	-4.7**	-3.9**	-3.4**	-0.2	-0.4	-0.9**
	[-5.3,-4.2]	[-4.6,-3.2]	[-4.1,-2.8]	[-0.7,0.3]	[-1.0,0.1]	[-1.4,-0.3]
Asian	-3.5**	-4.5**	-3.3**	-0.1	-0.3	-0.6
	[-4.2,-2.7]	[-5.4,-3.6]	[-4.0,-2.5]	[-0.8,0.6]	[-1.0,0.4]	[-1.3,0.1]
Race/ethnicity other than above	1.3**	1.3*	1.3**	0.1	-0.4	-1.1**
	[0.6,2.1]	[0.3,2.2]	[0.5,2.2]	[-0.6,0.9]	[-1.1,0.4]	[-1.9,-0.3]
Female	-2.1**	-3.9**	-3.4**	0.4+	0.4+	0.2
	[-2.5,-1.6]	[-4.5,-3.4]	[-3.9,-2.9]	[-0.1,0.8]	[-0.0,0.9]	[-0.3,0.7]
Expected source of payment: Medicare	-0.2	-2.3**	-1.2**	1.3**	1.5**	2.9**
	[-0.8,0.4]	[-3.1,-1.5]	[-1.9,-0.5]	[0.7,1.9]	[0.9,2.2]	[2.2,3.6]
Expected source of payment: Medicaid	0.6	-0.5	1.0**	2.3**	2.5**	3.4**
	[-0.1,1.3]	[-1.4,0.4]	[0.3,1.7]	[1.7,3.0]	[1.8,3.2]	[2.6,4.1]
Indigent: county or other	2.7**	1.3+	5.6**	-2.1**	-2.3**	-2.1**
	[1.6,3.7]	[-0.1,2.7]	[4.6,6.6]	[-2.6,-1.6]	[-2.8,-1.8]	[-2.7,-1.5]

© 2020 Hsia et al. *JAMA Network Open.*

eTable 1. Full regression results for Table 2 (continued)						
	**Admitted to PCI hospital**	**Receive PCI on the same day**	**Receive PCI during the episode**	**30-day mortality**	**90-day mortality**	**1-year mortality**
Expected source of payment: Patient	0.2	1.0+	1.6**	1.1**	1.1**	1.2**
	[-0.6,1.1]	[-0.0,2.1]	[0.8,2.5]	[0.5,1.7]	[0.5,1.7]	[0.5,1.8]
Expected source of payment: Other	4.1**	2.0*	2.9**	0.5	0.7	1.6**
	[3.1,5.2]	[0.5,3.5]	[1.7,4.0]	[-0.4,1.4]	[-0.3,1.7]	[0.5,2.7]
Age groups (reference <65)						
65-69	-0.6+	-0.5	-1.0**	2.2**	2.4**	2.2**
	[-1.4,0.1]	[-1.4,0.4]	[-1.8,-0.3]	[1.5,2.8]	[1.7,3.1]	[1.4,2.9]
70-74	-1.4**	-2.7**	-2.6**	4.3**	5.0**	5.7**
	[-2.2,-0.6]	[-3.7,-1.7]	[-3.5,-1.8]	[3.6,5.1]	[4.2,5.8]	[4.8,6.6]
75-79	-2.9**	-6.0**	-6.4**	8.0**	9.2**	10.5**
	[-3.7,-2.0]	[-7.1,-5.0]	[-7.3,-5.4]	[7.1,8.8]	[8.2,10.1]	[9.5,11.4]
80-84	-5.5**	-11.6**	-13.0**	12.2**	14.3**	16.8**
	[-6.4,-4.6]	[-12.7,-10.5]	[-14.0,-11.9]	[11.3,13.2]	[13.3,15.3]	[15.8,17.9]
85-99	-9.6**	-25.8**	-31.9**	22.9**	27.0**	32.0**
	[-10.5,-8.7]	[-26.8,-24.7]	[-32.9,-30.9]	[21.9,23.8]	[26.0,28.1]	[30.9,33.0]
**Patient comorbid conditions**						
Peripheral vascular disease	-0.3	-2.4**	-0.5	2.6**	3.3**	4.3**
	[-1.1,0.4]	[-3.3,-1.5]	[-1.3,0.3]	[1.8,3.4]	[2.5,4.1]	[3.4,5.2]
Pulmonary Circulation disorders	0.1	-7.0**	-3.6**	3.7**	5.2**	6.8**
	[-1.1,1.4]	[-8.5,-5.4]	[-5.0,-2.1]	[2.1,5.2]	[3.6,6.9]	[5.1,8.5]
Diabetes	-1.7**	-3.9**	-3.1**	2.5**	2.9**	4.1**
	[-2.1,-1.2]	[-4.4,-3.4]	[-3.5,-2.6]	[2.1,2.9]	[2.5,3.4]	[3.7,4.6]
Renal failure	-4.8**	-11.4**	-10.1**	6.4**	8.4**	11.8**
	[-5.5,-4.1]	[-12.2,-10.6]	[-10.9,-9.4]	[5.7,7.1]	[7.6,9.2]	[11.0,12.6]
Liver disease	-5.1**	-9.7**	-10.7**	5.6**	6.9**	7.9**
	[-6.9,-3.4]	[-11.7,-7.7]	[-12.6,-8.7]	[3.9,7.4]	[5.1,8.7]	[6.0,9.8]

© 2020 Hsia et al. *JAMA Network Open.*

eTable 1. Full regression results for Table 2 (continued)						
	**Admitted to PCI hospital**	**Receive PCI on the same day**	**Receive PCI during the episode**	**30-day mortality**	**90-day mortality**	**1-year mortality**
Cancer	-4.3**	-12.0**	-13.9**	13.8**	19.3**	26.6**
	[-5.7,-3.0]	[-13.5,-10.5]	[-15.4,-12.4]	[12.2,15.4]	[17.6,20.9]	[24.9,28.3]
Dementia	-2.2*	-12.4**	-15.8**	4.8**	6.7**	8.1**
	[-4.0,-0.5]	[-14.1,-10.6]	[-17.6,-14.0]	[2.7,6.8]	[4.6,8.8]	[5.9,10.2]
Valvular disease	2.3**	-2.8**	0.6	0.9*	2.2**	3.6**
	[1.6,3.1]	[-3.7,-1.9]	[-0.3,1.4]	[0.1,1.7]	[1.4,3.1]	[2.7,4.5]
Hypertension	1.1**	0.4	2.2**	-3.8**	-4.1**	-4.1**
	[0.7,1.5]	[-0.1,0.9]	[1.8,2.6]	[-4.2,-3.5]	[-4.5,-3.7]	[-4.6,-3.7]
Chronic pulmonary disease	-2.4**	-5.6**	-4.3**	1.1**	2.1**	3.8**
	[-2.9,-1.8]	[-6.2,-4.9]	[-4.9,-3.7]	[0.6,1.7]	[1.5,2.7]	[3.2,4.5]
Rheumatoid arthritis/collagen vascular	-0.5	-1.9*	-0.8	0.2	1.2	2.0*
	[-2.0,1.0]	[-3.7,-0.1]	[-2.4,0.9]	[-1.2,1.7]	[-0.4,2.8]	[0.3,3.7]
Coagulation deficiency	6.9**	4.8**	5.7**	6.6**	8.5**	8.5**
	[6.0,7.7]	[3.6,6.0]	[4.6,6.8]	[5.4,7.8]	[7.2,9.7]	[7.2,9.8]
Obesity	-0.8**	-1.4**	-0.2	-1.4**	-1.7**	-2.3**
	[-1.3,-0.2]	[-2.1,-0.7]	[-0.8,0.4]	[-1.8,-0.9]	[-2.2,-1.2]	[-2.8,-1.7]
Substance abuse	-2.4**	-5.2**	-3.9**	0.6	0.7+	1.0*
	[-3.2,-1.5]	[-6.3,-4.2]	[-4.8,-3.0]	[-0.1,1.3]	[-0.0,1.5]	[0.2,1.8]
Depression	-2.0**	-4.3**	-3.6**	-1.4**	-0.9+	-0.3
	[-2.9,-1.0]	[-5.4,-3.2]	[-4.7,-2.6]	[-2.3,-0.5]	[-1.9,0.0]	[-1.3,0.7]
Psychosis	-7.4**	-8.8**	-8.4**	-0.5	0.5	0.5
	[-8.9,-5.9]	[-10.4,-7.1]	[-10.0,-6.8]	[-1.8,0.8]	[-1.0,1.9]	[-1.0,2.1]
Hypothyroidism	-1.2**	-3.5**	-2.5**	-1.0*	-0.7+	-0.4
	[-2.0,-0.5]	[-4.4,-2.6]	[-3.4,-1.7]	[-1.7,-0.2]	[-1.6,0.1]	[-1.3,0.4]
Paralysis and other neurological disorder	-2.4**	-8.6**	-10.1**	11.4**	12.5**	14.1**
	[-3.3,-1.5]	[-9.6,-7.5]	[-11.1,-9.1]	[10.4,12.5]	[11.4,13.6]	[13.0,15.2]

© 2020 Hsia et al. *JAMA Network Open.*

eTable 1. Full regression results for Table 2 (continued)						
	**Admitted to PCI hospital**	**Receive PCI on the same day**	**Receive PCI during the episode**	**30-day mortality**	**90-day mortality**	**1-year mortality**
Chronic Peptic ulcer disease	-6.5	-0.9	-4.8	-1.7	-5.3	-4.7
	[-16.6,3.7]	[-12.5,10.8]	[-15.3,5.8]	[-9.5,6.2]	[-13.0,2.5]	[-13.9,4.4]
Weight loss	-6.3**	-9.8**	-9.8**	2.9**	9.1**	12.7**
	[-7.9,-4.7]	[-11.5,-8.1]	[-11.5,-8.2]	[1.2,4.7]	[7.2,11.0]	[10.8,14.6]
Fluid and electrolyte disorders	-0.6*	-5.9**	-5.7**	13.3**	14.5**	14.3**
	[-1.1,-0.0]	[-6.5,-5.2]	[-6.4,-5.1]	[12.6,13.9]	[13.8,15.2]	[13.6,15.0]
Anemia (blood loss and deficiency)	2.2**	-1.1**	0.5	-2.7**	-1.4**	0.7+
	[1.6,2.8]	[-1.8,-0.3]	[-0.2,1.2]	[-3.3,-2.1]	[-2.1,-0.7]	[-0.1,1.4]
Patient was a transfer	18.4**	24.2**	23.1**	-3.2**	-3.5**	-4.1**
	[18.1,18.8]	[23.6,24.8]	[22.8,23.5]	[-3.6,-2.7]	[-3.9,-3.0]	[-4.6,-3.6]
Year indicator (reference year is 2006)						
Year of admission=2007	2.4**	3.9**	2.4**	-0.5	-0.7+	-0.9*
	[1.5,3.3]	[3.0,4.9]	[1.5,3.3]	[-1.2,0.2]	[-1.4,0.0]	[-1.7,-0.1]
Year of admission=2008	3.9**	6.7**	4.2**	-0.9**	-1.5**	-1.8**
	[3.0,4.8]	[5.7,7.7]	[3.3,5.1]	[-1.6,-0.3]	[-2.2,-0.8]	[-2.6,-1.0]
Year of admission=2009	6.7**	10.2**	7.4**	-1.4**	-2.2**	-3.3**
	[5.8,7.6]	[9.1,11.2]	[6.5,8.3]	[-2.1,-0.7]	[-2.9,-1.5]	[-4.1,-2.5]
Year of admission=2010	8.1**	13.4**	9.9**	-1.4**	-2.3**	-3.4**
	[7.2,9.0]	[12.3,14.4]	[9.0,10.9]	[-2.2,-0.7]	[-3.0,-1.5]	[-4.2,-2.6]
Year of admission=2011	10.9**	16.5**	12.4**	-1.5**	-2.4**	-3.7**
	[10.0,11.8]	[15.5,17.6]	[11.5,13.4]	[-2.2,-0.7]	[-3.1,-1.6]	[-4.5,-2.9]
Year of admission=2012	11.3**	19.3**	14.6**	-1.8**	-2.6**	-3.4**
	[10.4,12.2]	[18.2,20.3]	[13.7,15.6]	[-2.6,-1.1]	[-3.4,-1.8]	[-4.3,-2.6]
Year of admission=2013	13.2**	20.2**	16.0**	-1.6**	-2.8**	-6.0**
	[12.3,14.1]	[19.1,21.3]	[15.0,17.0]	[-2.4,-0.8]	[-3.7,-2.0]	[-6.8,-5.1]
Year of admission=2014	14.7**	20.7**	16.8**			

© 2020 Hsia et al. *JAMA Network Open.*

eTable 1. Full regression results for Table 2 (continued)						
	**Admitted to PCI hospital**	**Receive PCI on the same day**	**Receive PCI during the episode**	**30-day mortality**	**90-day mortality**	**1-year mortality**
	[13.8,15.7]	[19.6,21.8]	[15.8,17.8]			
Year of admission=2015	13.3**	21.5**	17.9**			
	[12.3,14.3]	[20.3,22.7]	[16.9,19.0]			
Constant term	73.5**	62.1**	73.4**	4.6**	5.1**	6.3**
	[72.6,74.4]	[61.1,63.1]	[72.5,74.3]	[3.9,5.2]	[4.4,5.8]	[5.5,7.0]
N	135579	139257	139257	117896	117896	117896

Abbreviations: PCI – percutaneous coronary intervention
+p<0.10, * p<0.05, ** p<0.01

© 2020 Hsia et al. *JAMA Network Open.*

eTable 2. Full regression results for Figure 2						
	**Admitted to PCI hospital**	**Receive PCI on the same day**	**Receive PCI during the episode**	**30-day mortality**	**90-day mortality**	**1-year mortality**
**Changes in percentage point after community and individual is exposed to regionalization**						
White in non-minority com	6.5**	5.4**	5.5**	-0.8*	-0.9*	-1.0*
	[5.6,7.3]	[4.4,6.4]	[4.6,6.3]	[-1.6,-0.0]	[-1.8,-0.1]	[-1.9,-0.2]
Black or Hispanic in non-minority	7.3**	4.5**	3.6**	0.5	0.8	1.0
communities	[5.6,8.9]	[2.6,6.4]	[1.9,5.3]	[-0.8,1.9]	[-0.7,2.2]	[-0.5,2.6]
Other non-white in non-minority	4.2**	3.5**	3.1**	0.3	0.9	1.3+
communities	[2.6,5.7]	[1.6,5.3]	[1.5,4.7]	[-1.0,1.7]	[-0.5,2.3]	[-0.2,2.7]
White in minority communities	5.0**	2.6**	1.4*	-0.5	-0.5	-0.4
	[4.0,6.1]	[1.3,3.8]	[0.3,2.5]	[-1.4,0.5]	[-1.4,0.5]	[-1.4,0.7]
Black or Hispanic in minority	4.1**	0.8	-0.1	-0.2	-0.0	0.5
communities	[2.8,5.5]	[-0.8,2.3]	[-1.5,1.3]	[-1.2,0.9]	[-1.1,1.1]	[-0.8,1.7]
Other non-white in minority	3.3**	0.6	-0.2	0.1	1.0	1.5*
communities	[1.7,4.9]	[-1.2,2.5]	[-1.8,1.4]	[-1.3,1.4]	[-0.4,2.4]	[0.0,3.0]
Baseline differences b/w non-minority and minority communities	1.4**	0.3	1.9**	0.9*	1.0*	1.1*
	[0.4,2.3]	[-0.7,1.4]	[0.9,2.9]	[0.1,1.7]	[0.2,1.9]	[0.2,2.0]
**Patient characteristics**						
Race (reference: white)						
African American	-4.2**	-5.9**	-6.2**	-1.3*	-1.6**	-0.5
	[-5.7,-2.7]	[-7.5,-4.3]	[-7.7,-4.7]	[-2.4,-0.2]	[-2.8,-0.4]	[-1.8,0.8]
Hispanic	-4.4**	-2.7**	-2.2**	-0.7	-1.1*	-1.8**
	[-5.7,-3.1]	[-4.0,-1.3]	[-3.4,-0.9]	[-1.6,0.3]	[-2.1,-0.1]	[-2.8,-0.7]
Asian	-1.9**	-3.0**	-1.7*	-0.7	-1.6**	-2.2**
	[-3.3,-0.5]	[-4.6,-1.5]	[-3.1,-0.3]	[-1.8,0.4]	[-2.7,-0.5]	[-3.4,-1.0]
Other race/ethnicity	2.9**	2.7**	2.9**	-0.5	-1.6**	-2.7**
	[1.5,4.3]	[1.1,4.4]	[1.5,4.3]	[-1.6,0.6]	[-2.7,-0.5]	[-3.9,-1.5]

© 2020 Hsia et al. *JAMA Network Open.*

eTable 2. Full regression results for Figure 2 (continued)						
	**Admitted to PCI hospital**	**Receive PCI on the same day**	**Receive PCI during the episode**	**30-day mortality**	**90-day mortality**	**1-year mortality**
Female	-2.1**	-3.9**	-3.4**	0.4+	0.4+	0.2
	[-2.5,-1.6]	[-4.5,-3.4]	[-3.9,-2.9]	[-0.1,0.8]	[-0.0,0.9]	[-0.3,0.7]
Expected source of payment:	-0.2	-2.3**	-1.2**	1.3**	1.5**	2.9**
Medicare	[-0.8,0.5]	[-3.1,-1.5]	[-1.9,-0.5]	[0.7,1.9]	[0.9,2.2]	[2.2,3.6]
Expected source of payment:	0.6+	-0.5	1.0**	2.3**	2.5**	3.4**
Medicaid	[-0.1,1.3]	[-1.4,0.4]	[0.2,1.7]	[1.7,3.0]	[1.8,3.2]	[2.6,4.1]
Expected source of payment:	2.7**	1.3+	5.6**	-2.1**	-2.3**	-2.1**
Indigent: county or other	[1.6,3.7]	[-0.1,2.7]	[4.6,6.6]	[-2.6,-1.6]	[-2.8,-1.8]	[-2.7,-1.5]
Expected source of payment:	0.2	1.0+	1.6**	1.1**	1.1**	1.2**
Patient	[-0.6,1.1]	[-0.0,2.1]	[0.8,2.5]	[0.5,1.7]	[0.5,1.7]	[0.5,1.8]
Expected source of payment:	4.1**	2.0*	2.9**	0.5	0.7	1.6**
Other	[3.1,5.2]	[0.5,3.5]	[1.7,4.0]	[-0.5,1.4]	[-0.4,1.7]	[0.5,2.7]
Age groups (reference <65)						
65-69	-0.7+	-0.5	-1.1**	2.2**	2.4**	2.2**
	[-1.4,0.1]	[-1.4,0.4]	[-1.8,-0.3]	[1.5,2.8]	[1.7,3.1]	[1.4,3.0]
70-74	-1.4**	-2.7**	-2.6**	4.4**	5.0**	5.7**
	[-2.2,-0.6]	[-3.7,-1.7]	[-3.5,-1.8]	[3.6,5.1]	[4.2,5.8]	[4.8,6.6]
75-79	-2.9**	-6.0**	-6.4**	8.0**	9.2**	10.5**
	[-3.7,-2.0]	[-7.1,-5.0]	[-7.4,-5.4]	[7.1,8.9]	[8.3,10.1]	[9.5,11.4]
80-84	-5.5**	-11.6**	-13.0**	12.2**	14.4**	16.8**
	[-6.4,-4.6]	[-12.7,-10.5]	[-14.0,-12.0]	[11.3,13.2]	[13.4,15.4]	[15.8,17.9]
85-99	-9.6**	-25.8**	-31.9**	22.9**	27.0**	32.0**
	[-10.5,-8.7]	[-26.8,-24.7]	[-32.9,-30.9]	[21.9,23.8]	[26.0,28.1]	[30.9,33.0]
**Patient comorbid conditions**						
Peripheral vascular disease	-0.3	-2.4**	-0.5	2.6**	3.3**	4.3**
	[-1.1,0.4]	[-3.3,-1.5]	[-1.3,0.3]	[1.8,3.4]	[2.5,4.1]	[3.4,5.2]

© 2020 Hsia et al. *JAMA Network Open.*

eTable 2. Full regression results for Figure 2 (continued)						
	**Admitted to PCI hospital**	**Receive PCI on the same day**	**Receive PCI during the episode**	**30-day mortality**	**90-day mortality**	**1-year mortality**
Pulmonary Circulation disorders	0.2	-6.9**	-3.6**	3.7**	5.2**	6.8**
	[-1.1,1.4]	[-8.5,-5.4]	[-5.0,-2.1]	[2.1,5.2]	[3.6,6.9]	[5.1,8.5]
Diabetes	-1.7**	-3.9**	-3.1**	2.5**	2.9**	4.2**
	[-2.1,-1.3]	[-4.5,-3.4]	[-3.5,-2.6]	[2.1,2.9]	[2.5,3.4]	[3.7,4.6]
Renal failure	-4.8**	-11.4**	-10.1**	6.4**	8.4**	11.8**
	[-5.5,-4.1]	[-12.2,-10.6]	[-10.9,-9.4]	[5.7,7.1]	[7.7,9.2]	[11.0,12.6]
Liver disease	-5.1**	-9.7**	-10.6**	5.6**	6.9**	7.9**
	[-6.9,-3.3]	[-11.7,-7.7]	[-12.6,-8.7]	[3.9,7.4]	[5.1,8.7]	[6.0,9.8]
Cancer	-4.3**	-12.0**	-13.9**	13.8**	19.3**	26.6**
	[-5.6,-3.0]	[-13.5,-10.5]	[-15.4,-12.4]	[12.2,15.4]	[17.6,20.9]	[24.9,28.3]
Dementia	-2.2*	-12.3**	-15.8**	4.8**	6.7**	8.0**
	[-4.0,-0.4]	[-14.1,-10.6]	[-17.6,-14.0]	[2.7,6.8]	[4.6,8.8]	[5.9,10.1]
Valvular disease	2.3**	-2.8**	0.6	0.9*	2.2**	3.6**
	[1.6,3.1]	[-3.7,-1.9]	[-0.3,1.4]	[0.1,1.7]	[1.4,3.1]	[2.7,4.5]
Hypertension	1.1**	0.4	2.2**	-3.8**	-4.1**	-4.1**
	[0.7,1.5]	[-0.1,0.9]	[1.8,2.6]	[-4.2,-3.5]	[-4.5,-3.7]	[-4.6,-3.7]
Chronic pulmonary disease	-2.4**	-5.6**	-4.3**	1.1**	2.1**	3.8**
	[-2.9,-1.8]	[-6.2,-4.9]	[-4.9,-3.7]	[0.6,1.7]	[1.5,2.6]	[3.2,4.5]
Rheumatoid arthritis/collagen	-0.5	-1.8*	-0.7	0.2	1.2	2.0*
Vascular	[-2.0,1.0]	[-3.6,-0.0]	[-2.4,0.9]	[-1.2,1.7]	[-0.4,2.8]	[0.3,3.7]
Coagulation deficiency	6.9**	4.8**	5.7**	6.6**	8.5**	8.5**
	[6.0,7.7]	[3.6,6.0]	[4.6,6.8]	[5.4,7.8]	[7.2,9.7]	[7.2,9.8]
Obesity	-0.8**	-1.4**	-0.2	-1.4**	-1.7**	-2.3**
	[-1.4,-0.2]	[-2.1,-0.7]	[-0.8,0.4]	[-1.8,-0.9]	[-2.2,-1.2]	[-2.8,-1.7]
Substance abuse	-2.4**	-5.2**	-3.9**	0.6	0.7+	1.0*
	[-3.2,-1.5]	[-6.3,-4.2]	[-4.8,-3.0]	[-0.1,1.3]	[-0.0,1.5]	[0.2,1.8]

© 2020 Hsia et al. *JAMA Network Open.*

eTable 2. Full regression results for Figure 2 (continued)						
	**Admitted to PCI hospital**	**Receive PCI on the same day**	**Receive PCI during the episode**	**30-day mortality**	**90-day mortality**	**1-year mortality**
Depression	-2.0**	-4.3**	-3.7**	-1.4**	-0.9+	-0.3
	[-2.9,-1.0]	[-5.4,-3.2]	[-4.7,-2.6]	[-2.3,-0.5]	[-1.9,0.0]	[-1.3,0.7]
Psychosis	-7.4**	-8.8**	-8.4**	-0.5	0.5	0.5
	[-8.9,-5.9]	[-10.4,-7.2]	[-10.0,-6.8]	[-1.8,0.8]	[-1.0,1.9]	[-1.0,2.1]
Hypothyroidism	-1.2**	-3.5**	-2.6**	-1.0*	-0.7+	-0.4
	[-2.0,-0.5]	[-4.4,-2.6]	[-3.4,-1.7]	[-1.7,-0.2]	[-1.6,0.1]	[-1.3,0.4]
Paralysis and other neurological	-2.4**	-8.6**	-10.1**	11.4**	12.5**	14.1**
disorder	[-3.3,-1.5]	[-9.6,-7.5]	[-11.1,-9.1]	[10.4,12.5]	[11.4,13.6]	[13.0,15.2]
Chronic Peptic ulcer disease	-6.5	-0.9	-4.8	-1.6	-5.2	-4.6
	[-16.7,3.6]	[-12.6,10.8]	[-15.3,5.7]	[-9.5,6.2]	[-12.9,2.6]	[-13.8,4.5]
Weight loss	-6.3**	-9.8**	-9.8**	2.9**	9.1**	12.7**
	[-7.9,-4.7]	[-11.5,-8.1]	[-11.5,-8.2]	[1.2,4.7]	[7.2,11.0]	[10.8,14.5]
Fluid and electrolyte disorders	-0.6*	-5.9**	-5.7**	13.3**	14.5**	14.3**
	[-1.1,-0.0]	[-6.5,-5.2]	[-6.4,-5.1]	[12.6,13.9]	[13.8,15.2]	[13.6,15.0]
Anemia (blood loss and	2.2**	-1.1**	0.5	-2.7**	-1.4**	0.6+
deficiency)	[1.6,2.8]	[-1.8,-0.3]	[-0.2,1.2]	[-3.3,-2.1]	[-2.1,-0.7]	[-0.1,1.4]
Patient was a transfer	18.4**	24.2**	23.1**	-3.2**	-3.4**	-4.1**
	[18.0,18.8]	[23.6,24.8]	[22.8,23.5]	[-3.6,-2.7]	[-3.9,-3.0]	[-4.6,-3.5]
Year indicator (reference year is 2006)						
Year of admission=2007	2.4**	3.9**	2.4**	-0.5	-0.7+	-0.9*
	[1.5,3.3]	[3.0,4.9]	[1.5,3.3]	[-1.2,0.2]	[-1.4,0.0]	[-1.7,-0.1]
Year of admission=2008	3.9**	6.7**	4.2**	-0.9**	-1.5**	-1.8**
	[3.0,4.8]	[5.7,7.7]	[3.3,5.1]	[-1.6,-0.3]	[-2.2,-0.8]	[-2.6,-1.1]
Year of admission=2009	6.7**	10.2**	7.4**	-1.4**	-2.2**	-3.3**
	[5.8,7.6]	[9.2,11.2]	[6.5,8.3]	[-2.1,-0.7]	[-2.9,-1.5]	[-4.1,-2.5]

© 2020 Hsia et al. *JAMA Network Open.*

eTable 2. Full regression results for Figure 2 (continued)						
	**Admitted to PCI hospital**	**Receive PCI on the same day**	**Receive PCI during the episode**	**30-day mortality**	**90-day mortality**	**1-year mortality**
Year of admission=2010	8.1**	13.4**	9.9**	-1.4**	-2.3**	-3.4**
	[7.2,9.0]	[12.3,14.4]	[9.0,10.9]	[-2.2,-0.7]	[-3.0,-1.5]	[-4.2,-2.6]
Year of admission=2011	10.9**	16.5**	12.4**	-1.5**	-2.4**	-3.7**
	[10.0,11.8]	[15.5,17.6]	[11.5,13.3]	[-2.2,-0.7]	[-3.1,-1.6]	[-4.5,-2.9]
Year of admission=2012	11.3**	19.3**	14.6**	-1.8**	-2.6**	-3.4**
	[10.4,12.2]	[18.2,20.3]	[13.7,15.6]	[-2.6,-1.1]	[-3.4,-1.8]	[-4.3,-2.6]
Year of admission=2013	13.2**	20.2**	16.0**	-1.6**	-2.8**	-6.0**
	[12.3,14.2]	[19.1,21.3]	[15.0,17.0]	[-2.4,-0.9]	[-3.7,-2.0]	[-6.9,-5.1]
Year of admission=2014	14.8**	20.7**	16.8**			
	[13.8,15.7]	[19.6,21.8]	[15.8,17.8]			
Year of admission=2015	13.3**	21.5**	18.0**			
	[12.4,14.3]	[20.3,22.7]	[16.9,19.0]			
Constant term	73.3**	61.8**	73.2**	4.6**	5.3**	6.5**
	[72.4,74.2]	[60.8,62.8]	[72.2,74.1]	[4.0,5.3]	[4.5,6.0]	[5.7,7.3]
N	135579	139257	139257	117896	117896	117896

Abbreviations: PCI – percutaneous coronary intervention
+p<0.10, * p<0.05, ** p<0.01

© 2020 Hsia et al. *JAMA Network Open.*

eTable 3. Sensitivity analysis using propensity score methods to match counties with similar pre-regionalization mortality trend						
	**Admitted to PCI hospital**	**Receive PCI on the same day**	**Receive PCI during the episode**	**30-day mortality**	**90-day mortality**	**1-year mortality**
Baseline differences b/w non-minority and minority communities	1.7	0.5	2.5	0.8	0.8	0.6
95% CI	[0.6,2.7]	[-0.6,1.7]	[1.4,3.5]	[-0.0,1.6]	[-0.0,1.7]	[-0.3,1.5]
Changes in outcome after non-minority county is regionalized	6.7	5.5	5.4	-0.5	-0.6	-0.7
95% CI	[5.8,7.5]	[4.4,6.5]	[4.5,6.3]	[-1.3,0.3]	[-1.4,0.2]	[-1.6,0.2]
Additional change in outcome in minority communities relative to non-minority	-1.9	-3.2	-4.4	0.2	0.5	1.0
95% CI	[-3.0,-0.8]	[-4.4,-2.0]	[-5.5,-3.3]	[-0.7,1.1]	[-0.5,1.4]	[-0.0,2.0]
N	127965	131196	131196	111023	111023	111023

Abbreviations: PCI – percutaneous coronary intervention, CI – confidence interval

© 2020 Hsia et al. *JAMA Network Open.*

**eTable 4. Sensitivity analysis of main model controlling for admission to PCI hospital**					
	**Receive PCI on the same day**	**Receive PCI during the episode**	**30-day mortality**	**90-day mortality**	**1-year mortality**
Sample mean at baseline (%)	49.7%	64.2%	13.6%	16.6%	21.4%
Baseline differences b/w non-minority and minority communities	-0.4	1.1	0.9	0.9	0.8
95% CI	[-1.3,0.5]	[0.4,1.9]	[0.1,1.6]	[0.1,1.7]	[-0.0,1.7]
Changes in outcome after non-minority county is regionalized	1.2	0.4	-0.3	-0.3	-0.3
95% CI	[0.3,2.1]	[-0.3,1.1]	[-1.0,0.5]	[-1.1,0.4]	[-1.1,0.5]
Additional change in outcome in minority communities relative to non-minority	-2.1	-2.8	0.1	0.4	0.7
95% CI	[-3.1,-1.1]	[-3.6,-1.9]	[-0.7,1.0]	[-0.5,1.3]	[-0.2,1.7]
N	135579	135579	115046	115046	115046

Abbreviations: PCI – percutaneous coronary intervention, CI – confidence interval

© 2020 Hsia et al. *JAMA Network Open.*

eTable 5. Sensitivity analysis excluding 410.9x						
	**Admitted to PCI hospital**	**Receive PCI on the same day**	**Receive PCI during the episode**	**30-day mortality**	**90-day mortality**	**1-year mortality**
Sample mean at baseline (%)	72.7%	49.7%	64.2%	13.6%	16.6%	21.4%
Baseline differences b/w non-minority and minority communities	1.2	0.2	1.9	0.7	0.7	0.8
95% CI	[0.2,2.2]	[-0.9,1.4]	[0.9,2.9]	[-0.1,1.4]	[-0.1,1.5]	[-0.1,1.7]
Changes in outcome after non-minority county is regionalized	5.9	5.2	4.5	-0.2	-0.3	-0.5
95% CI	[5.1,6.8]	[4.2,6.3]	[3.6,5.3]	[-1.0,0.5]	[-1.1,0.5]	[-1.3,0.4]
Additional change in outcome in minority communities relative to non-minority	-1.4	-2.9	-3.7	0.3	0.6	0.7
95% CI	[-2.4,-0.3]	[-4.2,-1.7]	[-4.7,-2.6]	[-0.5,1.1]	[-0.3,1.4]	[-0.2,1.7]
N	112406	115297	115297	97468	97468	97468

Abbreviations: PCI – percutaneous coronary intervention, CI – confidence interval

© 2020 Hsia et al. *JAMA Network Open.*

eTable 6. Sensitivity analysis using alternative definition of PCI

	**Broader definition**		**Narrower definition**	
	**Receive PCI on the same day**	**Receive PCI during the episode**	**Receive PCI on the same day**	**Receive PCI during the episode**
Sample mean at baseline (%)	49.7%	64.2%	40.9%	49.1%
Baseline differences b/w non-minority and minority communities	0.6	2.2	1.1	1.2
95% CI	[-0.4,1.7]	[1.3,3.2]	[0.1,2.2]	[0.2,2.3]
Changes in outcome after non-minority county is regionalized	5.1	5.0	5.4	5.6
95% CI	[4.2,6.1]	[4.2,5.9]	[4.4,6.4]	[4.7,6.6]
Additional change in outcome in minority communities relative to non-minority	-3.4	-4.3	-3.7	-3.5
95% CI	[-4.5,-2.2]	[-5.3,-3.2]	[-4.8,-2.5]	[-4.6,-2.4]
N	139257	139257	139257	139257

Abbreviations: PCI – percutaneous coronary intervention, CI – confidence interval

© 2020 Hsia et al. *JAMA Network Open.*

eTable 7. Sensitivity analysis using alternative definition of minority community						
	**Admitted to PCI hospital**	**Receive PCI on the same day**	**Receive PCI during the episode**	**30-day mortality**	**90-day mortality**	**1-year mortality**
Sample mean at baseline (%)	72.7%	49.7%	64.2%	13.6%	16.6%	21.4%
Baseline differences b/w non-minority and minority communities	2.1	1.7	2.5	1.1	1.3	1.3
95% CI	[1.1,3.1]	[0.6,2.8]	[1.5,3.5]	[0.3,1.8]	[0.5,2.1]	[0.4,2.2]
Changes in outcome after non-minority county is regionalized	6.4	4.9	4.6	-0.5	-0.5	-0.5
95% CI	[5.6,7.2]	[4.0,5.9]	[3.8,5.4]	[-1.2,0.2]	[-1.2,0.3]	[-1.3,0.3]
Additional change in outcome in minority communities relative to non-minority	-2.6	-4.0	-4.4	0.2	0.3	0.6
95% CI	[-3.7,-1.6]	[-5.2,-2.7]	[-5.5,-3.3]	[-0.7,1.0]	[-0.7,1.2]	[-0.3,1.6]
N	135579	139257	139257	117896	117896	117896

Notes: Minority community is defined as a ZIP code that is in top quartile of Black or Hispanic share of population.
Abbreviations: PCI – percutaneous coronary intervention, CI – confidence interval

© 2020 Hsia et al. *JAMA Network Open.*

eFigure. Regression-adjusted percentage point changes in outcomes by minority status at individual and community levels after exposure to regionalization when controlling for access to PCI capable hospitals

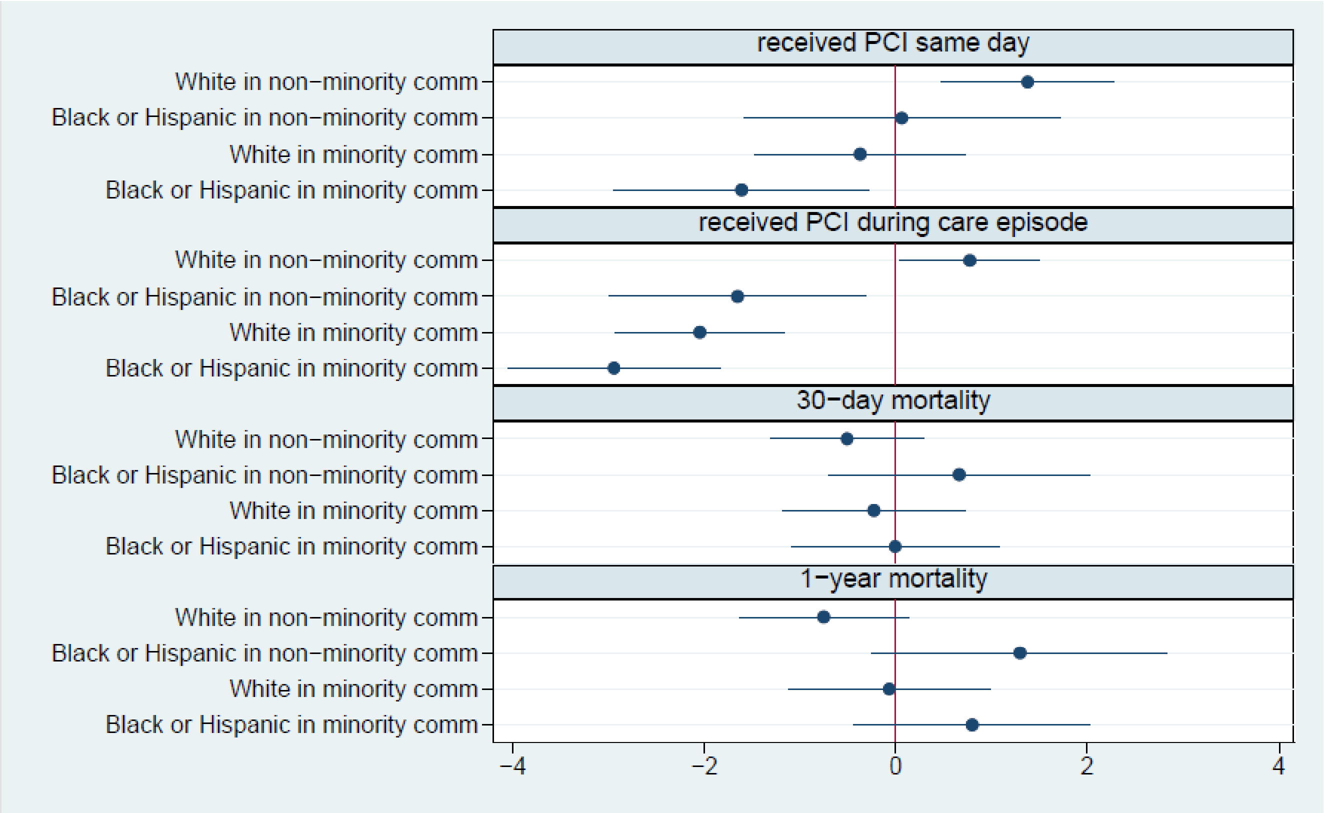

Abbreviations: PCI – percutaneous coronary intervention

© 2020 Hsia et al. *JAMA Network Open.*
